# Biological and clinical aspects of HPV-related cancers

**DOI:** 10.20892/j.issn.2095-3941.2020.0370

**Published:** 2020-12-15

**Authors:** Klaudia Anna Szymonowicz, Junjie Chen

**Affiliations:** 1 Department of Experimental Radiation Oncology, The University of Texas MD Anderson Cancer Center, Houston, TX 77030, USA

**Keywords:** Human papillomavirus (HPV), cervical cancer, HNC, DNA repair, clinical trials, cancer statistics

## Abstract

Cancer-related diseases represent the second overall cause of death worldwide. Human papilloma virus (HPV) is an infectious agent which is mainly sexually transmitted and may lead to HPV-associated cancers in both men and women. Almost all cervical cancers are HPV-associated, however, an increasing number of head and neck cancers (HNCs), especially oropharyngeal cancer, can be linked to HPV infection. Moreover, anogenital cancers, including vaginal, vulvar, penial, and anal cancers, represent a subset of HPV-related cancers. Whereas testing and prevention of cervical cancer have significantly improved over past decades, anogenital cancers remain more difficult to confirm. Current clinical trials including patients with HPV-related cancers focus on finding proper testing for all HPV-associated cancers as well as improve the currently applied treatments. The HPV viral oncoproteins, E6 and E7, lead to degradation of, respectively, p53 and pRb resulting in entering the S phase without G1 arrest. These high-risk HPV viral oncogenes alter numerous cellular processes, including DNA repair, angiogenesis, and/or apoptosis, which eventually result in carcinogenesis. Additionally, a comprehensive analysis of gene expression and alteration among a panel of DNA double strand breaks (DSB) repair genes in HPV-negative and HPV-positive HNC cancers reveals differences pointing to HPV-dependent modifications of DNA repair processes in these cancers. In this review, we discuss the current knowledge regarding HPV-related cancers, current screening, and treatment options as well as DNA damage response-related biological aspects of the HPV infection and clinical trials.

## Cancer statistics worldwide and in the United States

**Figure 1:**
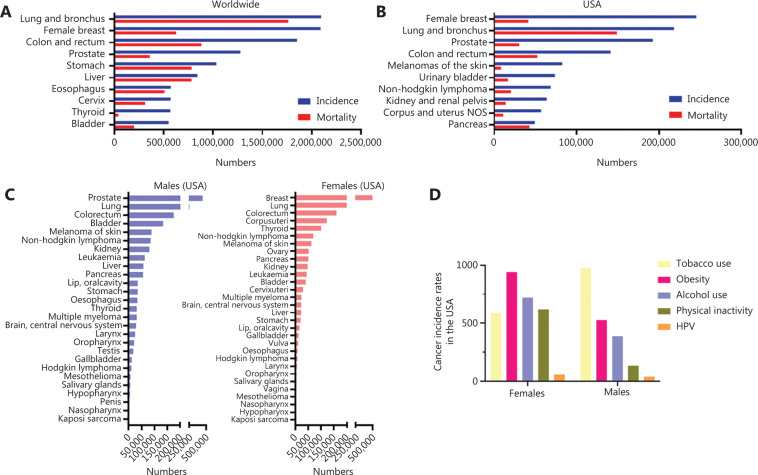
Cancer incidence and mortality numbers according to the WHO and the Centers for Disease Control (CDC). (A) Incidence (blue) and mortality (red) numbers for 10 most prominent cancer types worldwide. (B) Incidence (blue) and mortality (red) numbers for 10 most prominent cancer types in the US. (C) Number of cancer cases in the US in men (left) and women (right) according to the CDC. (D) Rates for cancer associated with the most common five risk factors in women and men. Tobacco use-associated cancers include: oral cavity and pharynx, esophagus; stomach; colon and rectum; liver; pancreas; larynx; lung, bronchus, and trachea; cervix; kidney and renal pelvis; urinary bladder; and acute myeloid leukemia. Obesity-associated cancers include adenocarcinoma of the esophagus; cancers of the breast (in postmenopausal women), colon and rectum, endometrium (corpus uterus), gallbladder, gastric cardia, kidney (renal cell), liver, ovary, pancreas, and thyroid; meningioma, and multiple myeloma. Alcohol-associated cancers include: oral cavity and pharynx, esophagus, colon and rectum, liver, larynx, and female breast cancer. Physical inactivity-associated cancers include breast cancer in postmenopausal women, endometrium (corpus uterus) cancer, and colon cancer. HPV-associated cancers include microscopically confirmed carcinoma of the cervix and squamous cell carcinomas of the vagina, vulva, penis, anus, rectum, and oropharynx.

Cancer-related diseases represent the second leading cause of death worldwide and in the United States (US) behind cardiovascular diseases and stroke, and led to around 9.6 million deaths globally in 2018^1^. As of 2016, the latest year for which incidence data are available, 1,658,716 new cases of cancer were reported, and 598,031 people died of cancer in the US. According to the American Cancer Society (ACS), more than 1.8 million new cases of cancer are estimated for 2020^3^. Worldwide, lung, breast, and colorectum cancer show the highest numbers of new cases; on the other hand, the highest mortality is caused by lung, colorectum, stomach, and liver cancer^1^ (**Figure 1A**). In the US, the cancer type-related incidence rate resembles the global pattern with most new cases for breast and lung cancers and the highest mortality in patients suffering from lung, colorectum, and pancreas cancers^2^ (**Figure 1B**). Importantly, the high incidence of lung and colorectal cancers was very high in both men and women^2^ (**Figure 1C**). Fortunately, the overall cancer-associated death rate in the US has dropped by 29% since 1991 saving more than 2.9 million lives, as of 2017^3^. This remarkable result is due to prevention and education, early detection, and the enormous improvement in cancer treatment. Although awareness across the population increased, the behavioral factors like alcohol use, unhealthy diet, and/or lack of physical activity, or tobacco use are still the most relevant cancer-associated risk factors^4^. Of note, tobacco use causes the majority of diverse cancer types with approximately 22% of all cancer deaths worldwide^1^. Interestingly, in the US the distribution of cancer-related risk factors is gender-dependent^2^ (**Figure 1D**). In men, the most cancer deaths were related to tobacco use, whereas in women most were related to obesity followed by alcohol use and poor physical activity^2^ (**Figure 1D**). Besides these lifestyle and diet-related risk factors, infection agents like the human papilloma virus (HPV) may lead to HPV-associated cancers in both men in women. In this review, we strive to give an overview of the available statistics, current testing and treatment options, clinical trials, and we also discuss the biological aspects of the HPV infection.

## HPV-associated cancers and current statistics

**Figure 2:**
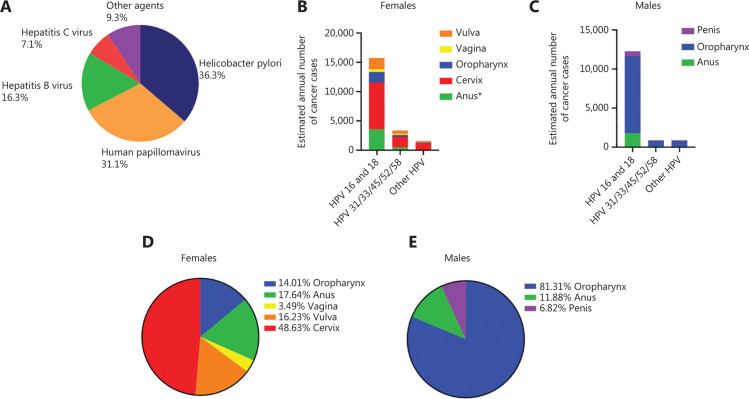
HPV-associated cancer types in men and women according to the WHO and the CDC. (A) Distribution of infection-caused cancers according to infectious agents worldwide in men and women. (B) Numbers of cancers caused by distinct HPV subtypes in women. (C) Numbers of cancers caused by distinct HPV subtypes in men. (D) Percentage of different HPV-associated cancers in women. (E) Percentage of different HPV-associated cancers in men.

Despite the diversity of cancer-related risk factors, infections with bacteria and viruses are known risk factors for cancer. Worldwide, the most prominent infectious agents causing cancer are *Helicobacter pylori* (36.3%) and HPV (31.1%), followed by hepatitis B (16.3%) and C (7.1%)^4^ (**Figure 2A**). Despite lower numbers of cancer related to infection with other agents, including Epstein–Barr virus (EBV), human herpesvirus type 8 (HHV-8; also known as Kaposi sarcoma-associated herpesvirus), and human T-cell lymphotropic virus type 1 (HTLV-1) as well as three parasites: *Opisthorchis viverrini*, *Clonorchis sinensis*, and *Schistosoma haematobium*, are a cause of nearly 10% of all infection-mediated cancers (**Figure 2A**).

HPVs belong to the family *Papillomaviridae*. The virons are non-enveloped and contain a double-stranded DNA genome. The genetic material is enclosed by an icosahedral capsid composed of major and minor structural proteins, L1 and L2, respectively. These viruses are highly tissue-specific and infect both cutaneous and mucosal epithelium. Based on the genomic sequence of L1, the gene encoding the principal capsid protein, over 200 HPV types have been identified and characterized^5^, with at least 14 high risk types which may cause cancer^6^. Two HPV types (16 and 18) cause the most HPV-related cancers and about 70% of cervical cancers and pre-cancerous cervical lesions^6^ (**Figure 2B, C**). Importantly, HPV is mainly transmitted through sexual activity but people can also be infected by skin-to skin contact^6^. Notably, about 80% of people – both men and women – will get a HPV infection at some point in their lives. All sexually active persons can be infected with low- or high-risk HPV and usually become infected with HPV shortly after the onset of sexual activity. However, most often the immune system clears the virus, but for some, the infection persists and can lead to precancerous changes. In more detail, cells infected with high-risk HPV increase their proliferation rate and, over time, if not controlled by the immune system, they may form precancerous changes or a tumor. Factors like long-term usage of oral contraceptives, many pregnancies and smoking, a compromised immune system and coinfection with another sexually transmitted disease as well as the HPV type (high risk) may increase the risk of precancerous cervical cells leading to cancer^2^. Of note, nearly all cervical cancer cases are related to HPV infection. Nevertheless, HPV infection can be linked to other types of cancer, including anogenital cancer (vulvar, vaginal, anal, and penial), head and neck cancers (HNCs) or genital warts in both men and women. The estimated median annual incidence of new anogenital warts is 137 per 100,000 men and 121 per 100,000 women and the prevalence ranges from 0.15% to 0.18% in men and women, respectively^7,8^. In general, HPV is thought to be responsible for more than 90% of anal and cervical cancers, about 70% of vaginal and vulvar cancers, and more than 60% of penile cancers. In contrast, oropharyngeal cancers traditionally have been caused by tobacco and alcohol, but recent studies show that about 70% of cancers of the oropharynx may be linked to HPV. Many cancers of the oropharynx may be caused by a combination of tobacco, alcohol, and HPV infection^2^. Worldwide, high-risk HPVs cause about 5% of all cancer cases, with an estimated number of infections of 570,000 in women and 60,000 in men each year. As of 2018, approximately 311,000 women died from cervical cancer worldwide and more than 85% of these deaths occurred in low- and middle-income countries where cervical cancer represents the leading cancer-related death in women^1^. The reason being that in these parts of world there is very limited access to screening tests, which would highly increase chances of early detection and allow more timely application of treatment^1^. In the US, high-risk HPVs cause 3% of all cancers in women and 2% of all cancers in men, and about 44,000 new cases of HPV infection-associated cancers are found annually, and about 34,800 of these cancers are terminal^2^. Importantly, nearly 50% of all HPV-associated cancers in women are cervical cancers, whereas in men oropharyngeal cancer was mostly related to HPV infection^9^ (**Figure 2D, E**). In males, the highest prevalence of becoming infected with a HPV was observed in HIV-infected men who have sex with other men^10,11^. Across heterosexual men, the highest risk of infection was found in African men having at least three female sexual partners^12^. In addition, the average prevalence of infection with any type of HPV was significantly higher in HIV-positive vs HIV-negative men, 78.2% vs 49.4%, respectively^13^.

## Current screening and treatment strategies for HPV-associated cancers

### Prevention and screenings

Comprehensive cervical cancer control includes primary prevention (vaccination against HPV), secondary prevention (screening and treatment of pre-cancerous lesions), tertiary prevention (diagnosis and treatment of invasive cervical cancer), and palliative care. Vaccines that protect against HPV 16 and 18 are recommended by the World Health Organization (WHO) and have been approved for use in many countries. All males and females ages 9–26 years should get the HPV vaccine. It is most effective when given at ages 11–12 years. The vaccine is given in two doses for males and females ages 9–14 years. Beginning at age 15 through age 45, three doses are required for full immunity. The HPV vaccine prevents HPV-related genital warts, most cervical, anal, vaginal, and vulvar cancers and reduces the risk of most HPV-related cancers of the throat and the penis. These vaccines consist of recombinant viral vaccine proteins containing purified virus-like particles for each virus genotype, thus they protect against different HPV types. Importantly, they are free of viral DNA so they are inactive and cannot infect cells. Three vaccines, Gardasil 4v (Merck&Co, Kenilworth, NJ, US), Gardasil 9v (Merck&Co, Kenilworth, NJ, US), and Cervarix 2v (GlaxoSmithKline Biologicals, Rixensart, Belgium), are now being marketed in many countries throughout the world – a quadrivalent, a nonavalent, and a bivalent vaccine, respectively. Both Gardasil vaccines are produced in recombinant *Saccharomyces cerevisiae,* whereas for Cervarix’s production the recombinant baculovirus expression system is applied^14^. The nonavalent provides additional protection against HPV types 31, 33, 45, 52, and 58. Data from clinical trials and initial post-marketing surveillance conducted in several continents show all three vaccines to be safe. However, rare cases of anaphylaxis (1.7 cases in 1 million doses), a life threatening allergic reaction, have been reported^14,15^. HPV-related post-vaccination syndromes with focus on the adjuvants, which increase the immune response to a certain antigen, have been investigated. The autoimmune/inflammatory syndrome Induced by adjuvants (AIAS) have been reported in 3.6 cases per 100,000 vaccine doses^16,17^. Moreover, it remains controversial if the rare death cases after HPV vaccine could be linked to the adjuvant or the vaccine itself^16,17^. Despite the rare cases of post-vaccination syndromes, HPV vaccines are reported as being very safe and are the most effective preventive option. However, HPV vaccine is not recommended in pregnant women because of a lack of scientific proof from clinical studies. Nevertheless, previous clinical trials including in pregnant women showed no increase in spontaneous abortions if compared to non-pregnant women^18^.

Most importantly, HPV vaccine is not protective against all types of HPV and therefore women still need to be screened despite previous vaccinations. It is recommended for women between the ages of 21 and 29 years to be Papanicolaou (Pap) tested every 3 years. For women between the ages of 30 and 65 years, additional HPV testing or HPV/Pap testing every 5 years is recommended. Women older than 65 years who were frequently tested and diagnosed negative may be advised to no longer be screened for cervical cancer. However, continuing screening is recommended if recent tests were abnormal. Whereas Pap tests screen for precancerous and cancerous processes in the cervix, HPV tests check for the occurrence of the HPV in the tissue. It is important to consider that a positive test after years of negative testing is not necessarily caused by a new infection but can be a result of virus reactivation years later. Currently, it is not possible to distinguish between a new infection and viral reactivation, nor can we compare the risk of both to cause cancer. A different approach of testing for cervical cancer is currently under investigation within a clinical trial which proposes using self-collected menstrual blood for HPV DNA and HPV E6/E7 mRNA analysis^19^ (NCT03638427).

Currently, screening can only be performed in cervical tissue as the U.S. Food and Drug Administration (FDA)-approved tests for other types of HPV-related cancers are not available yet. Nevertheless, an ongoing study is testing anal Pap smears and conducting high resolution anoscopy (HRA) to find a proper testing method for anal cancer^19^ (NCT03061435). Additionally, a current clinical study measures the amount of HPV DNA in blood samples from patients who have recently been diagnosed with cervical cancer, trying to correlate the DNA amount of HPV with the stage of disease or the applied treatment^19^ (NCT03749720). This approach may help to uncover a potential recurrence earlier than is currently possible.

Patients with HPV-positive HNC were previously described as younger, White, non-smokers with a high number of sexual partners^20,21^. However, the incidence among older and non-White people has increased according to other studies^22,23^. Indeed, a recent comprehensive study investigated the differences in HPV genotype distribution in an age-dependent manner showing higher levels of HPV in younger HPV-positive oropharynx cancer patients in comparison to older patients^6^. Interestingly, 100% of the younger individuals were HPV-positive in comparison to 76% among the older tested patients. The investigators claim that the HPV-positivity in oropharynx cancer depends on the intensity of sexual exposure explaining the lower numbers of HPV-positive cancers in older patients. HNC can include oral cavity, larynx, pharynx, paranasal sinuses, and nasal cavity and salivary glands, so the symptoms can vary. Nevertheless, most of the HNCs cause a sore throat that does not heal, problems with swallowing and changes in the voice. Depending on the specific location, it may also include difficulties in breathing, pain in the neck area, headaches, earache or chronic sinus infections^6^. While nowadays the number of HNC cases has decreased due to reduced use of alcohol and/or tobacco, the HPV infection-related incidence has increased^6^. Since the symptoms may be very dependent on the localization of the cancerous changes, different diagnostic tests may be necessary. Additionally, to confirm a cancerous lesion, a tissue sample needs to be examined under a microscope. To confirm a HPV infection, the National Comprehensive Cancer Network (NCCN) guidelines note that p16 protein expression is highly correlated with HPV status and recommend assessment of p16 expression by immunohistochemistry (IHC) or detection of HPV DNA in tumor cell nuclei by *in situ* hybridization (ISH)^6^.

### Treatment and its effectiveness

Presumably, access to Pap tests and HPV vaccine contributed to a decline in cervical cancer cases over decades which was also associated with a lower mortality^6^. According to the ACS^6^, the 5-year overall survival for cervical cancer is relatively high. Of note, HPV infection is not treatable but therapies for HPV-related precancerous cellular alternations and cancers are available. In most women with precancerous lesions, the loop electrosurgical excision procedure (LEEP) is applied^6^. During LEEP, the abnormal cells can be removed using an electrical current. Moreover, laser therapy, cryotherapy – freezing the abnormal tissue, and cold knife conization are the treatment options for precancerous lesions. External genital warts caused by HPV infection can be treated by topical medicine^7^.

As the testing for HNC depends on the type and location of the HNC, the treatment is also not unified for all HNCs, but it may include surgery, radiotherapy, chemotherapy, targeted therapy, or a combination of those^6^. A new type of surgery, transoral robotic surgery, is supported by three-dimensional (3D)-imaging which helps to remove tumors at places that are difficult to reach. Stereotactic body radiation applies high doses of radiation into the tumor by positioning the patient in the same way in each session which reduces the chance of damaging surrounding healthy tissue. Intensity-modulated radiation therapy (IMRT) uses 3D-imaging to precisely irradiate the tumor, which also reduces damage to healthy tissue. For oropharyngeal cancer, cetuximab, a monoclonal antibody for epidermal growth factor receptor (EGFR), which stops the tumor cell proliferation, is applied as a targeted therapy for HNC patients^6^. However, patients with HPV-related cancers receive the same treatment as patients with HPV-unrelated cancers at the same anatomical site. An FDA-approved anti-PD-1 antibody, pembrolizumab, became a first-line treatment option for all PD-L1 expressing tumors including HPV-positive and -negative HNC^24,25^. It can be used as a monotherapy or in combination with chemotherapy.

Because HPV-associated HNC respond vastly better to treatment than the HPV-negative ones, they also show a better survival rate. Using data obtained from the Surveillance, Epidemiology, and End Results HPV Status Database, a recent study reported that from 2013 to 2014, the US incidence of HPV-positive oropharyngeal squamous cell carcinoma (OPSCC) was 4.62 [95% confidence interval (CI), 4.51–4.73] vs 1.82 (95% CI, 1.75–1.89) per 100,000 persons for HPV-negative OPSCC and the incidence of HPV-positive vs negative non-OPSCC of the head and neck was 0.62 (95% CI, 0.58–0.66) vs 1.38 (95% CI, 1.32–1.44)^26^. Current treatments for both cervical cancer and HNC cover a wide spectrum of options, which not only kill the tumor but also consider the protection of healthy tissue.

### Biology of HPV-associated cancers and potentially underlying mechanisms in HPV infection

**Figure 3:**
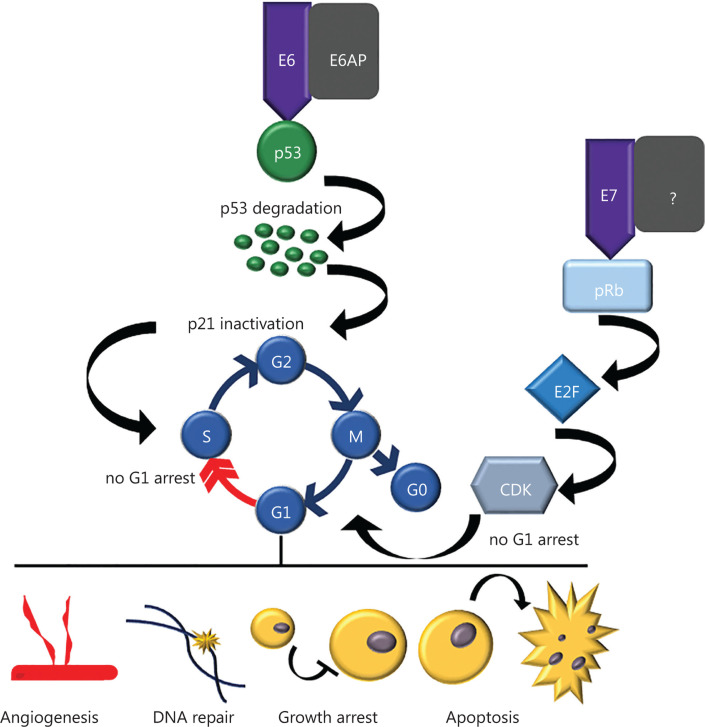
Effect of HPV E6 and E7 viral oncoproteins on cellular processes. E6 and E7 viral oncoproteins can affect angiogenesis, DNA repair, growth arrest and apoptosis by impairing G1 arrest. E6 binds to E6AP, and subsequently binds to p53 and promotes its degradation by ubiquitination. Thus, the function of p53 as a tumor suppressor is diminished and cell cycle is not properly controlled by p21, a cyclin-dependent kinase inhibitor that ensures G1/S checkpoint. E7 leads to degradation of pRb, the retinoblastoma protein that functions as a tumor suppressor. Because of RB inhibition by E7, cells can enter S phase without a G1 arrest.

Persistent cervical HPV infections may progress towards premalignant glandular or squamous intra-epithelial lesions, classified histopathologically as cervical intraepithelial neoplasia (CIN), and to cancer. CIN is further classified as: CIN 1: mild dysplasia; CIN 2: moderate to marked dysplasia; and CIN 3: severe dysplasia to carcinoma *in situ*. Most CIN lesions regress spontaneously, although over a number of years, lesions on the cervix can slowly become cancerous^27,28^. A diagnosis of CIN is based primarily on the presence of nuclear atypia and loss of normal squamous maturation (polarity). Accurate grading of CIN lesions is essential as the treatment and clinical follow-up algorithms are very different for the low-grade (CIN 1) and high-grade lesions (CIN 2 and 3)^29^. Although the likelihood of progression clearly increases with the increasing grade of cervical intraepithelial neoplasia, a proportion of high-grade lesions could still regress. Infection with high-risk HPV subtypes has been shown to be a risk factor for persistent and/or progressive cervical dysplasia. It has also been proposed that HPV DNA integration into host DNA is critical in cervical carcinogenesis^30,31^ mediated by disruption of the E1/E2 open reading frames of the HPV genome and subsequent loss of the E2-controlled regulation of E6 and E7^31^. This leads to strongly decreased expression levels of p53 in HPV-associated cancer cells^32^. The inactivation of the host p53 and retinoblastoma protein (pRb) by E6 and E7, respectively, results in suppressed cell cycle checkpoints and uncontrolled cell proliferation^33–36^ (**Figure 3**). In detail, E6 binds p53 leading to its degradation *via* E6-associated protein (E6AP)-mediated ubiquitination^33–37^. Interestingly, E6 first needs to bind to the protein E3-ligase E6AP as neither E6 or E6AP can bind to p53 alone^34,38^. Additionally, only high-risk HPV E6 and E7 can bind to p53 or pRb^39^ confirming that HPV-related cancers originate only from high-risk HPV types. Degradation of p53 leads to inactivation of one of its targets – p21 (also known as p21WAF^1^/Cip^1^)^40^, a cyclin-dependent kinase inhibitor, which prevents cells from entering the S phase by promoting cell cycle arrest in the G1 phase in response to many stimuli^41–44^ (**Figure 3**). E7 targets pRb for ubiquitination, leading to the release of E2F transcription factors, which transcribe cyclin E, cyclin A, and p16INK4A, an inhibitor of CDK4/6, forcing the cells through premature S phase entry^45^ (**Figure 3**). To date, it remains unclear how E7 mediates the pRb ubiquitination. However, it has been proposed that Cullin2 can bind to E7 and be responsible for the ubiquitination of pRb^46^. Of note, E7 was found to be ubiquitinated by the complex Cullin 1/Skp2^47^. Although pRb also interacts with Skp2, it is not involved in its ubiquitination^47^. Overall, degradation of both p53 and pRb mediated by high-risk HPV results in cellular immortalization which presumably leads to the development of high-grade dysplasia (CIN 2 and 3), with a potential to progress to invasive carcinoma^27,48^.

A recent *in vitro* report suggested applying antisense HPV RNA transcripts in combination with cisplatin for HPV-positive HNC cells^49^. Here, the investigators infected a HPV-positive HNC cell line, UM-SCC-47 harboring HPV 16, and the HPV-negative HNC cancer cell line YCU-T892, with antisense RNA transcripts of HPV 16 E6/E7 using a recombinant adenoviral vector Ad-E6/E7-AS. They observed a significantly suppressed cell growth in the HPV-positive but not in the HPV-negative cells which correlated with increased expression of p53 and pRb in the HPV-positive cell line. Finally, a combinatory treatment with cisplatin evoked a cell growth reduction *in vitro* and suppressed tumor growth significantly if compared to monotherapy of either cisplatin or transfection with an Ad-E6/E7-AS construct. Another study also reported suppressed cell growth in HPV-related cervical cancer cells after downregulating HPV 16 E6 and E7 using an adenovirus-mediated transfection which correlated with higher expression of p53 and/or pRb proteins^50,51^. Additionally, a similar effect of increased sensitization to cisplatin treatment has been shown in cervical cancer cells after treatment with siRNA against the HPV 16 and HPV 18 E6 and E7^52^. Furthermore, downregulation of HPV 16 and HPV 18 E6 and E7 led to radiosensitizing effects and suppressed growth in cervical cancer cells and xenograft mouse tumors^53^. Targeting HPV 16 E6/E7 by a gRNA induced apoptosis in HPV-positive cervical cancer cells SiHa and an additional treatment with a gRNA against PD1 significantly reduced the growth of SiHa cell-xenografted humanized SCID mice^54^. Taken together, downregulation of HPV E6 and E7 significantly suppresses cell growth and reconstitutes p53 and pRb expression. Moreover, downregulation of HPV makes tumor cells more susceptible to chemotherapy.

## Ongoing clinical trials on treatment for HPV-related cancers

Immunotherapy is nowadays listed as a treatment option for cancer patients. On the one hand, immunotherapy can be used as a booster in the form of checkpoint inhibitors or vaccines to trigger a patient’s immune system. On the other hand, immunotherapy uses monoclonal engineered T-cells or antibodies as substances mimicking the compounds of the immune system. Of note, the FDA has approved 14 different immunomodulators including seven checkpoint inhibitors disturbing the PD-1/PD-L1 interaction or blocking the CTLA-4 which can support the immune system to recognize the cancer cells and to induce cell death^55^. Moreover, four cytokines, small molecular messengers between cells, are approved for the treatment of patients with kidney cancer, leukemia, lymphoma, melanoma, and sarcoma. Since 1997 the FDA has approved multiple monoclonal antibodies for the treatment of several solid tumors and hematological cancers^56^. For HPV-related cancers, cervical and HNC, an anti-CTLA4 monoclonal antibody ipilimumab is being tested within phase II clinical studies alone or in combination with radiotherapy^19^ (NCT03799445, NCT01693783). CUE-101 is a novel E7-pHLA-IL-2-Fc fusion protein and its Fc domain is linked to the protein of interest. Thus, it is designed to activate and expand a population of tumor specific T cells to eradicate HPV-driven malignancies. It is currently being tested in a new phase I clinical study recruiting patients to verify the toxicity and estimate the optimal dosage^19^ (NCT03978689). Avelumab is a monoclonal antibody which targets protein programmed death-ligand 1 (PDL-1) and has been recently approved by the FDA for a specific type of skin cancer – Merkel cell carcinoma, renal, and bladder cancer. Currently, over 200 clinical trials are testing avelumab’s effect on different types of cancer. One of these trials combines avelumab with a newly developed vaccine TG4001 with HPV 16 antigens in HPV-related cancers^19^ (NCT03260023). Durvalumab is another FDA-approved human immunoglobulin G1 kappa (IgG1κ) monoclonal antibody which blocks the interaction of PD-L1 with the PD-1 and is used in treatment of different types of lung and bladder cancers. Durvalumab is now being tested with a vaccine MEDI0457 in HPV-positive OPSCC in a phase 2 clinical trial^19^ (NCT04001413). MEDI0457 is composed of DNA plasmids encoding the E6/E7 genes of HPV 16 and HPV 18 (VGX-3100) and DNA plasmid encoding the human interleukin-12 (IL-12), INO-9012, which triggers the immune system^6^. A recent paper revealed that MEDI0457 was well-tolerated by patients with cervical cancer after receiving chemoradiation and raised the idea of combining tumor-specific vaccines with radiotherapy^57^. MEDI0457 was also well tolerated by patients suffering from HPV-associated HNC cancer showing elevated antigen-specific T cell activity^58^. However, despite the promising safety studies, the final efficacy of a vaccine can only be evaluated after phase 2 and 3 clinical trials. Moreover, durvalumab is also being tested in combination with radiation in patients with HPV-associated OPSCC^19^ (NCT03623646).

Many kinase inhibitors have now entered cancer treatment. A phosphoinositide-3-kinase gamma (PI3Kγ)-inhibitor, IPI-549, is currently under investigation in a phase II clinical study including HNC cancer patients either as a monotherapy^19^ (NCT03795610) or in combination with PD-1 blocking antibody nivolumab in a panel of HPV-related cancers^19^ (NCT02379520). The underlying working hypothesis is that mRNA signatures of immune response will be increased in IPI-549-treated patients and therefore this combination may enhance anti-PD-1 therapy.

Interestingly, a novel treatment involving HPVST cells, HPV-specific T cells, is currently under investigation in one clinical study including patients with recurring HPV-related cancers^19^ (NCT02379520). HPVST cells originate from the patients who previously suffered from HPV-related cancer. The goal is to validate whether these cells can affect the tumors and whether they can develop resistance against transforming growth factor-beta (TGF-β) produced by HPV-mediated tumors. After the optimal dose of HPVST cells is established, an FDA-approved human IgG4 monoclonal antibody, nivolumab, that blocks PD-1, will be applied in combination with HPVST. The overall purpose of this study is to potentially increase the effectiveness of HPVST cells using the nivolumab in patients suffering from HPV-related cancers. A 6-month application of nivolumab is a final step of a phase II study including patients diagnosed with HPV-positive OPSCC who will first receive chemotherapy (carboplatin, nab-paclitaxel and nivolumab) followed by radiotherapy or surgery^19^ (NCT03107182, NCT03829722).

Another phase I clinical study uses magnetic resonance imaging (MRI)-guided brachytherapy (internal radiation therapy) to verify whether this approach can improve the high-risk clinical target volume (HR-CTV) D90 (dose to 90% of the high-risk clinical target volume) rate compared to conventional guidance using ultrasound and a freehand technique in stage IB2-IV cervical or stage II-IVA vaginal cancer^19^ (NCT03634267). Furthermore, proton therapy has attracted attention and is now being considered as treatment option for many cancer patients and can be applied in a variety of tumor types. An ongoing clinical trial is using the traditional radiotherapy and/or proton therapy in patients with HPV-related OPSCC to achieve a reduction of toxicity by maintaining the efficiency of the treatment^19^ (NCT03621696). The limitation of radiotherapy-induced side effects in patients suffering from HPV-associated HNC is a focus of a phase II studies applying IMRT and chemotherapy^19^ (NCT02281955, NCT01932697).

CRISPR/Cas9 technology is now widely used for *in vitro* and *in vivo* studies. The usage of this genome editing tool is currently under investigation in a phase I clinical trial^19^ (NCT03747965). In more detail, the clinical study includes patients with different solid tumors, especially pancreatic cancer, cholangiocarcinoma cancer, and ovarian cancer, and monitors the safety of CRISPR-Cas9-mediated PD-1 gene-knocked out chimeric antigen receptor (CAR) T cells. Another study is addressing the safety and efficacy of CRISPR/Cas9-HPV E6/E7, which can disrupt the DNA of HPV 16 and HPV 18, in patients with HPV-related cervical intra-epithelial neoplasia (CIN)^19^ (NCT03057912). Zinc finger nucleases, called ZFN-603 and ZFN-758, cleave HPV 16 and HPV 18 E7 oncogene and are currently under investigation including in patients suffering from CIN^19^ (NCT02800369).

Altogether, there are many ongoing clinical trials including patients suffering from HPV-related cancers which address different aspects and methods of treatment, but these trials mainly focus on unique HPV antigens for vaccination as well as in combination with immune checkpoint agents.

### *In silico* analysis-based correlations within HPV-associated cancers

A comprehensive analysis of 228 primary cervical cancers revealed an upregulation of *SHKBP1*, *ERBB3*, *CASP8*, *HLA-A*, and *TGFBR2* as novel significantly mutated genes in cervical cancer and confirmed mutations in *PIK3CA*, *EP300*, *FBXW7*, *HLA-B*, *PTEN*, *NFE2L2*, *ARID1A*, *KRAS*, and *MAPK1* in these patients^59^. In contrast, HPV-negative cancers, mostly including HNC and a few endometrial-like cervical cancers, showed an increased mutation rate in *ARID1A*, *KRAS*, and *PTEN*. Moreover, amplifications in *PD-L1* and *PD-L2*, and the BCAR4 long non-coding RNA which has previously been linked to respond to the chemotherapy drug lapatinib, an EGFR/HER2 inhibitor, have also been observed^59^. Importantly, over 70% of cervical cancers exhibited genomic alterations in either one or both of PI3K–MAPK and TGF-β signaling pathways, suggesting compounds of these pathways may be targets for anticancer therapy. Moreover, a cross-cancer analysis in this study highlighted the similarity among HPV-positive squamous cancers [cervical and HPV-related head and neck squamous cell carcinoma (HNSCC)] independent of the anatomical site. Interestingly, HPV 18-associated tumors exhibited a significantly higher ratio of unspliced vs spliced versions of the E6 protein than the HPV 16-related cancers showing the diversity between different HPV types. This suggests different functional aspects of E6 and E7 in tumors infected with different HPV types. Taken together, this study provides a comprehensive analysis of gene deregulations in cervical cancers including the dependence on HPV subtypes which underlines the importance of targeted therapy for certain cancer associated with particular HPV subtypes.

**Figure 4:**
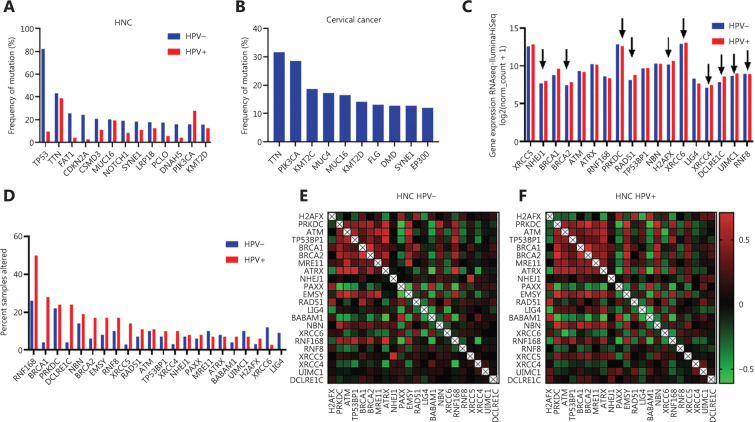
Different frequencies in gene mutations in HPV-positive and negatives cancers. (A) Frequency of mutations in HPV-positive and HPV-negative HNC cancer patients based on TCGA bioportal *in silico* analysis. (B) Frequency of mutations in cervical cancer patients based on TCGA bioportal *in silico* analysis. (C, D) Co-expression of DNA DSB repair genes in HPV-negative (C) and HPV-positive (D) HNC patients based on TCGA bioportal *in silico* analysis.

Our own *in silico* analysis including HPV-positive and HPV-negative HNC revealed that *TP53* was the most frequently mutated (over 80% of samples) gene in HPV-negative HNC^60^ (**Figure 4A**). In contrast, HPV-positive HNC samples were mostly harboring a mutation in *TTN* which encodes the protein Titin responsible for the passive elasticity of muscles. The second highly mutated gene was *PIK3CA* which correlates with findings of Burk^59^. Additionally, we performed the same analysis with cervical cancer samples and found that *TTN* and *PIK3CA* were also highly mutated in these samples (**Figure 4A, B**).

As DNA repair processes in tumor cells are affected, we performed an additional mRNA expression analysis including DNA repair genes within HPV-positive (*n* = 38) and HPV-negative (*n* = 75) HNC^61^. Our data reveals significant mRNA expression differences in *NHEJ1, BRCA2, PRKDC, RAD51, H2AFX, XRCC6, XRCC4, DCLRE1C, UIMC1*, and *RNF8* (**Figure 4C**). Interestingly, most of the genes, except for *PRKDC* and *RNF168*, showed higher expression in HPV-positive HNC. However, 50% of all HPV-positive HNC samples were altered in the *RNF168* gene which overlaps with a study from Sitz and colleagues^62^ (**Figure 4D**). Our analysis uncovered additionally high rates of alterations in the *BRCA1* gene in HPV-positive HNC samples (**Figure 4D**). As BRCA1 and RNF168 both promote homologous recombination repair (HRR)^63,64^, the high alteration rate of both suggests a HPV-mediated facilitated HRR in HPV-associated HNC cancers. In contrast, HPV-negative HNC samples showed alterations in the *LIG4* and *XRCC6* genes involved in non-homologous end joining (NHEJ), suggesting an affected DNA double strand breaks (DSB) repair by NHEJ (**Figure 4D**). However, comparing the co-expression of DNA DSB repair genes in HPV-positive and HPV-negative HNC has not shown striking differences^60^ (**Figure 4E, F**), indicating that co-expression between those genes cannot be significantly affected by the virus, despite the affected DNA repair processes described.

### Role of DNA repair in the treatment of HPV-mediated cancers

DNA damaging agents leading to generation of diverse lesions, for example, DNA DSBs, induce DNA damage response and DNA repair. DSBs represent the most severe type of DNA lesions repaired by NHEJ or HRR, which enforce the maintenance of genomic stability. NHEJ is a fast but error-prone DNA repair pathway with DNA-dependent protein kinase (DNAPKcs) as a key protein kinase important for recruitment of downstream factors necessary to join and ligate the broken DNA ends^65^. Proper DNA repair ensures cell survival and finally the health of an individual. However, different diseases, especially cancer, can result in imbalanced DNA damage response and repair, but also HPV infections result in the deregulations of DNA repair processes. DNAPKcs has been found to be downregulated in HPV-positive OPSCC if compared to HPV-negative carcinomas^66^ which we also observed in our *in silico* analysis (**Figure 4C**). Moreover, the decreased expression of DNAPKcs inversely correlated with the increased expression of the viral oncoproteins E6 and E7, and was associated with higher radiosensitivity^66^. Furthermore, cervical cancer cell lines and HPV-positive HNSCC, but not the HPV-negative HNSCC, were associated with higher amount of persistent infrared (IR)-induced γH2AX, a marker for DNA DSBs indicating a HPV-dependent impaired removal of DNA DSBs^66–68^. In line with this observation, it has been suggested that the lack of DNAPK and BRCA2 in HPV-positive HNSCC has led to a reduced DNA repair capacity by NHEJ and/or HRR, despite sustained activity of 53BP1 and BRCA1, which are independent of HPV status^66^. Moreover, it has been proposed that HPV decreases the efficacy of HRR by hindering RAD51 to localize to DNA DSBs^69^. A new report presented that E6-transduced U2OS cells failed to show colocalization of the Fanconi anemia complementation group D2 (FancD2),a protein involved in interstrand crosslink repair, with DNA DSBs, which subsequently hindered the recruitment of the downstream repair protein RAD51 to DSBs^70^. Further, E6 expression caused delayed FancD2 de-ubiquitination, an important process for effective interstrand crosslink repair, resulting in its increased chromatin retention. Finally, E6 could suppress USP1 de-ubiquitinating activity, and led to persistent activation of ATR/CHK-1/pS565 FancI signaling^70–72^. Thus, a prediction to sensitivity to DNA crosslinking agents in HPV-negative HNSCC based on an interstrand crosslink repair gene expression has been introduced and confirmed that defects in these DNA repair genes correlated with a poor prognosis^71^. Indeed, ATR or CHK1 inhibitors suppress both amplification and late viral gene expression in differentiated cells and reduce stable copy numbers of the viral genome in undifferentiated cells^73^. Moreover, application of the ATR inhibitor in both HPV- and HPV+ HNSCC cell lines led to enhanced sensitivity to cisplatin treatment^74^. Interestingly, ATM- and ATR-mediated signaling is constitutively active in HPV-associated cells unexposed to any DNA damaging agent^71,75,76^, suggesting that application of ATR and ATM inhibitors could represent a promising approach in the treatment of HPV-related carcinoma.

As the viral oncoproteins, E6 and E7, are crucial in HPV-mediated tumorigenesis, researchers are focusing on their mechanistic functions, partially in DNA repair, in promoting cancer. A recent study proposed E7 as the oncoprotein which hampers the DNA damage response to DSBs by interacting with the E3 ligase RNF168 involved in DNA DSB repair^62^. It has also been observed that expression of an exonuclease with a proofreading function, Three prime repair exonuclease 1 (TREX1), is upregulated exclusively in HPV-transformed primary keratinocytes expressing HPV 16 E6 and E7^77^. Downregulation of TREX1 resulted in inhibition of cell growth, which was associated with p53 upregulation.

Remarkably, HPV infection can block TGF-β resulting in more prominent activation of alternative end joining (altEJ) instead of HRR, enhancing the cellular sensitivity to cisplatin or PARP inhibitors^78–80^ suggesting that HPV-positive cancer cells are more sensitive to therapy by interfering with DNA DSB repair. In line with this observation, HPV-positive OPSCCs are more sensitive to radiotherapy than the HPV-negative ones, correlating with the reduced DNA DSB repair capacity in HPV-positive cancers^81^. Several studies showed that HPV-positive HNSCC cells treated with ionizing radiation show a significantly higher cell death than the HPV-negative HNSCC^82–85^. Importantly, a higher radiosensitizing effect of olaparib was observed in HPV-negative OPSCCs than in HPV-positive OPSCC, suggesting this combined treatment to be applied in relatively more radioresistant HPV-negative cancers^85^. In addition, roscovitine, a cyclin-dependent kinase inhibitor targeting CDK2, CDK7, and CDK9, has been proposed as an olaparib-enhancer towards radiosensitization for HPV-negative HNSCC^86^. In contrast, a study using a panel of HPV-positive and HPV-negative OPSCC revealed that only a subset of HPV-positive OPSCCs were susceptible for olaparib treatment at a therapeutic concentration (0.1–0.5 μM), whereas other HPV-positive and HPV-negative OPSCCs were either sensitive to the PARP inhibition at higher doses or not at all^87^.

The wide spectrum of cancer treatment includes proton beam irradiation which uses particles to irradiate tumors. Despite a relatively low number of studies including proton irradiation and HPV-related cancers, most reports claim an advantage of proton beam therapy especially for relatively radioresistant HPV-negative cancers. For instance, proton irradiation-induced reduction of cell growth in HPV-negative HNC cells was significantly higher than the standard photon-based radiation^88,89^. Furthermore, a comprehensive analysis of protein expression in HPV-positive and HPV-negative HNC cell lines revealed different expression patterns between proton and photon irradiation^90^. In brief, they found differences in expression of IL-6, GSK3, Src, AMPK, cyclooxygenase (COX)-2, vascular endothelial growth factor (VEGF) pathways in HNC cells treated with proton or photon irradiation^90^, suggesting varying benefit from both radiotherapy modulations in combination with targeted therapy or immunotherapy.

On the other hand, HPV infection can activate different DNA repair proteins involved in response to ultraviolet (UV)-mediated DNA damage, including nucleotide end resection (NER), the Fanconi anemia (FA) pathway, and translesion synthesis (TLS) because HPV requires these DNA repair mechanisms to properly replicate its genome^71,75,76,91,92^. However, it has also been shown that HPV 31 needs an accumulation of ATM and HR-related factors, for example, MRN complex composed of RAD50-MRE11-NBS1 proteins, to its replication sites^75,76,92^. Furthermore, HPV abolishes the apoptosis upon agents inducing UV-mediated lesions^93–95^ suggesting that HPV can induce resistance to DNA crosslinking agents, for example, cisplatin. To predict a potential cisplatin resistance in cervical cancer, different proteins have been identified, for example, p53^96–98^, excision repair cross-complementation group 1 (ERCC1)^99^, an apoptosis regulator B-cell lymphoma 2 (Bcl-2), p21^97,98,100^. This approach supports screenings and personalized treatment for HPV-related cancers to avoid resistances and to apply the best suitable treatment following diagnosis.
